# TNFAIP3 is required for FGFR1 activation-promoted proliferation and tumorigenesis of premalignant DCIS.COM human mammary epithelial cells

**DOI:** 10.1186/s13058-018-1024-9

**Published:** 2018-08-15

**Authors:** Mao Yang, Xiaobin Yu, Xuesen Li, Bo Luo, Wenli Yang, Yan Lin, Dabing Li, Zhonglin Gan, Jianming Xu, Tao He

**Affiliations:** 1 Institute for Cancer Medicine and School of Basic Medical Sciences, Southwest Medical University, Luzhou, Sichuan 646000 China; 2 Department of Molecular and Cellular Biology, Baylor College of Medicine, Houston, TX 77030 USA

**Keywords:** Breast cancer, FGFR1, Gene regulation, TNFAIP3, Cell proliferation, Tumor growth

## Abstract

**Background:**

Although ductal carcinoma in situ (DCIS) is a non-invasive breast cancer, many DCIS lesions may progress to invasive cancer and the genes and pathways responsible for its progression are largely unknown. FGFR1 plays an important role in cell proliferation, differentiation and carcinogenesis. The purpose of this study is to examine the roles of FGFR1 signaling in gene expression, cell proliferation, tumor growth and progression in a non-invasive DCIS model.

**Methods:**

DCIS.COM cells were transfected with an empty vector to generate DCIS-Ctrl cells. DCIS-iFGFR1 cells were transfected with an AP20187-inducible iFGFR1 vector to generate DCIS-iFGFR1 cells. iFGFR1 consists of the v-Src myristoylation membrane-targeting sequence, FGFR1 cytoplasmic domain and the AP20187-inducible FKBP12 dimerization domain, which simulates FGFR1 signaling. The CRISPR/Cas9 system was employed to knockout *ERK1*, *ERK2* or *TNFAIP3* in DCIS-iFGFR1 cells. Established cell lines were treated with/without AP20187 and with/without FGFR1, MEK, or ERK1/2 inhibitor. The effects of these treatments were determined by Western blot, RNA-Seq, real-time RT-PCR, cell proliferation, mammosphere growth, xenograft tumor growth, and tumor histopathological assays.

**Results:**

Activation of iFGFR1 signaling in DCIS-iFGFR1 cells enhanced ERK1/2 activities, induced partial epithelial-to-mesenchymal transition (EMT) and increased cell proliferation. Activation of iFGFR1 signaling promoted DCIS growth and progression to invasive cancer derived from DCIS-iFGFR1 cells in mice. Activation of iFGFR1 signaling also altered expression levels of 946 genes involved in cell proliferation, migration, cancer pathways, and other molecular and cellular functions. TNFAIP3, a ubiquitin-editing enzyme, is upregulated by iFGFR1 signaling in a FGFR1 kinase activity and in an ERK2-dependent manner. Importantly, TNFAIP3 knockout not only inhibited the AP20187-induced proliferation and tumor growth of DCIS-iFGFR1 cells, but also further reduced baseline proliferation and tumor growth of DCIS-iFGFR1 cells without AP20187 treatment.

**Conclusions:**

Activation of iFGFR1 promotes ERK1/2 activity, EMT, cell proliferation, tumor growth, DCIS progression to invasive cancer, and altered the gene expression profile of DCIS-iFGFR1 cells. Activation of iFGFR1 upregulated TNFAIP3 in an ERK2-dependent manner and TNFAIP3 is required for iFGFR1 activation-promoted DCIS.COM cell proliferation, mammosphere growth, tumor growth and progression. These results suggest that TNFAIP3 may be a potential target for inhibiting DCIS growth and progression promoted by FGFR1 signaling.

**Electronic supplementary material:**

The online version of this article (10.1186/s13058-018-1024-9) contains supplementary material, which is available to authorized users.

## Background

The high incidence of breast cancer is a severe threat to woman’s health [1]. Ductal carcinoma in situ (DCIS) is the earliest detectable form of breast cancer, which represents 20–25% of newly diagnosed breast cancers [2, 3]. Although DCIS contains malignant tumor cells confined within the basement membrane and is non-lethal, about 14–50% of DCIS cases are estimated to progress to invasive cancer over time if left untreated [4]. To date, there are still no histopathological classification or conventional biomarkers that can accurately predict whether a DCIS lesion will progress to invasive and metastatic breast cancer. The molecular mechanisms responsible for DCIS progression are also largely unclear. The cell lines derived from the MCF10A normal human breast epithelial cells exhibit different grades of malignancy, which have been used as cellular models for studying breast cancer progression, including DCIS progression. Specifically, MCF10AT cells were derived from MCF10A cells transfected with mutated T24 Ha-ras that carries a G12D mutation [5]. The original transplants of MCF10AT cells in mice mainly generated differentiated ducts lined by simple or hyperplastic epithelium. Serial passages of the MCF10AT xenografts produced different grades of lesions that recapitulated the human proliferative breast disease in most mice, as well as DCIS and invasive cancer in a small subset of mice [6, 7]. DCIS.COM is a clonal breast cancer cell line derived from a passaged MCF10AT xenograft with DCIS morphology [8]. Injection of DCIS.COM cells into SCID mice produces rapidly growing lesions that are predominantly comedo DCIS [8]. This DCIS.COM model has been successfully used for studying DCIS progression in vivo [8, 9]. In this study, we utilize the DCIS.COM model to study the impact of fibroblast growth factor receptor 1 (FGFR1) signaling on DCIS growth and progression.

The FGFR tyrosine kinase family with FGFR1/2/3/4 plays important roles in cancer [10]. Whole genome sequencing data of multiple types of human cancers showed that amplifications, mutations and rearrangements of FGFR1/2/3/4 were detected in 3.5%, 1.5%, 2.0%, and 0.5%, respectively [11]. Importantly, the frequency of these genetic aberrations of FGFR1 was found to be particularly high in breast cancers, which reached 18% of the breast tumor samples examined [11]. FGFRs and their ligands, fibroblast growth factors (FGFs), also promote breast cancer resistance to endocrine therapy and chemotherapy [12–14]. Therefore, it is important to understand how FGF and FGFR signaling pathways promote breast cancer growth and progression.

There are 22 FGFs in human, and 18 of these FGFs can bind to the extracellular domains of one or more FGFRs in the presence of heparan sulfate and/or Klotho co-receptors [15–18]. In addition to their extracellular ligand-binding domain, FGFRs also contain transmembrane and intracellular tyrosine kinase domains. Upon FGF binding, FGFR dimerizes and transphosphorylates specific tyrosine residues in each intracellular domain of the dimer, resulting in activation of downstream signaling pathways via direct or indirect interactions with FGFR substrate 2α (FRS2α), PLCγ and/or STAT1/3/5. The FGFR-phosphorylated FRS2α further relays the signal to activate the Ras-Raf-MEK-ERK1/2 pathway [17, 19]. The FGF-FGFR signaling pathways play crucial roles in cell growth, cell differentiation, embryonic development, and many physiological processes [10]. Their activities are subjected to precise temporal and spatial regulatory mechanisms, while their deregulations may cause many severe developmental and physiological health problems [20].

Abnormal activation of FGF/FGFR signaling pathways can increase cell proliferation, induce epithelial-to-mesenchymal transition (EMT), cell motility and invasiveness, promote carcinogenesis, and make cancer cell resistant to drug treatment [10]. For example, although activation of the FGF signaling in the estrogen receptor-positive (ER+) MCF7 breast cancer cells is unable to enhance cell proliferation [21], activation of FGFR1 in the ER-negative (ER-) human mammary epithelial cells increases cell proliferation, and knockdown of FGFR1 in the ER- mouse breast cancer cells also inhibits cell proliferation [22, 23]. Deregulated FGFR1 signaling also causes epithelial hyperplasia or adenocarcinoma and synergizes with the Wnt1-signaling pathway or PTEN loss-activated PI3K-AKT signaling to drive carcinogenesis and metastasis in mouse models of breast or prostate cancers [24, 25]. FGFR1 amplification and overexpression in certain breast cancer cells increases MAPK and PI3K-AKT activities [26]. In ER+/human epidermal growth factor receptor 2-negative (HER2-) breast cancers, FGFR gene amplification is more frequent in endocrine therapy-resistant cases versus endocrine therapy-sensitive cases [12]. Human breast tumors with FGFR1 overexpression possess higher cell proliferation rates and have poor prognosis [26]. It has also been shown that HER2 expression in breast cancer cells upregulates FGF2 and FGFR1, which promotes EMT and resistance to Lapatinib [14]. Moreover, certain cancer cells resistant to paclitaxel or EGFR, Met and VEGFR inhibitors can regain sensitivity to these drugs after blocking the FGF/FGFR signaling [27–30]. Finally, although clinical trials with FGFR inhibitors are currently underway, it is possible that FGFR mutation, gene fusion, alternative kinase activation or MAPK/Akt reactivation may make the cancer cells resistant to these inhibitors [31–34]. Given all these detrimental roles of FGF/FGFR signaling pathways in promoting carcinogenesis and possible resistance of cancer cells to FGFR inhibitors, it is important to find alternative molecular targets of the FGF/FGFR signaling through identifying their regulated genes important for this signaling pathway-promoted carcinogenesis.

Based on molecular mechanisms of FGFR1 activation by FGF, a ligand-inducible chimeric FGFR1 (iFGFR1) fusion protein has been created to mimic the FGF/FGFR1 signaling system [35, 36]. This fusion protein consists of the v-Src myristoylation membrane-targeting sequence, the cytoplasmic domain of FGFR1 for signaling and two repeats of the AP20187-inducible FKBP12 dimerization domain. AP20187-induced dimerization of this iFGFR1 fusion protein faithfully activates the FGFR1 signaling pathway [35, 36]. In the current study, we have generated iFGFR1-expressing DCIS.COM cell lines and used these cell lines as a model to study the impact of FGFR1 signaling on the growth, progression and gene expression of breast DCIS tumor cells. We show that the AP20187-activated iFGFR1 enhances extracellular-signal regulated kinases 1/2 (ERK1/2) MAPK activities, increases DCIS.COM cell proliferation in culture and promotes DCIS progression to invasive cancer in mice. Activation of iFGFR1 in DCIS.COM cells altered the expression levels of many genes involved in cancer and other cellular functions. Among the iFGFR1-upregulated genes, we are particularly interested in TNFAIP3. TNFAIP3 is a ubiquitin-editing enzyme with both deubiquitylase and E3 ubiquitin ligase activity [37]. Multiple studies have reported tumor suppressor roles for TNFAIP3 in inhibiting NF-κB in chronic myeloid leukemia [38], suppressing EMT, cell migration and invasion in nasopharyngeal carcinoma [39], and inhibiting liver inflammation, hepatocellular carcinoma proliferation, and metastasis through inhibition of *Twist1* expression and TNFα-induced cell motility [40]. However, other studies have reported the cancer-promoting roles for TNFAIP3 in conferring tamoxifen resistance in ER+ breast cancers [41], promoting EMT and metastasis of basal-like breast cancers by mono-ubiquitination of SNAIL1 [42], and preventing adult T-cell leukemia cells from apoptosis [43]. TNFAIP3 has also been found to be overexpressed in metastatic cholangiocarcinomas and esophageal squamous cell carcinomas [44, 45]. In the current study, we found that iFGFR1 activation upregulates TNFAIP3 expression through activating ERK2 MAPK in DCIS.COM cells. We also demonstrate that knockout (KO) of TNFAIP3 blocks FGFR1 signaling-promoted DCIS cell proliferation and progression, suggesting that TNFAIP3 is required for FGFR1 signaling-promoted DCIS growth and progression.

## Methods

### Plasmids, cell lines and cell culture

pSH1/M-FGFR1-Fv-Fvls-E plasmid for iFGFR1 expression was provided by Dr. David M. Spencer [25]. The iFGFR1 DNA sequence in this plasmid was subcloned into the pRevTRE plasmid to generate the pRevTRE-iFGFR1 plasmid. DCIS.COM cells were cultured in DMEM/F12 (1:1) medium with 5% horse serum, 29 mM sodium bicarbonate, 10 mM HEPES, 100 IU/ml penicillin and 100 μg/ml penicillin/streptomycin (PS) as described previously [9]. PT67 cells were cultured in DMEM with 10% fetal bovine serum (FBS) and PS. All cells were cultured at 37 °C in an incubator supplied with 5% CO_2_.

### Generation of iFGFR1-expressing cell lines

PT67 cells (2 × 10^6^) were cultured overnight and then transfected with 5 μg of pRevTRE or pRevTRE-iFGFR1 plasmids using Lipofectamine 3000 Reagent (Invitrogen, Waltham, MA, USA). The transfected cells were cultured in the medium containing 400 μg/ml of hygromycin for 2 weeks. The conditioned medium of the transfected PT67 cells containing retrovirus particles was filtered through a 0.45 μm membrane, and then used to transduce DCIS.COM cells for 24 h in the presence of 4 μg/ml polybrene. These cells were growth-selected in medium containing 400 μg/ml of hygromycin for 2 weeks. Surviving clones were picked up and expanded for immunoblotting using an HA antibody to detect the iFGFR1 C-terminal HA tag. Clones expressing iFGFR1 were designated as DCIS-iFGFR1 cell lines. Clones transduced by pRevTRE empty virus served as DCIS control (DCIS-Ctrl) cells.

### Cell growth assay

DCIS-Ctrl, DCIS-iFGFR1, and TNFAIP3 KO DCIS-iFGFR1 cells were seeded in 96- or 6-well plate at 2 × 10^3^ or 10^5^ cells/well, cultured overnight, and treated with 0.02% DMSO (vehicle) or 100 nM AP20187 for different time periods. CellTiter method was used to measure cell viability. In this assay, 20 μl of CellTiter 96 Aqueous One Solution (Promega, Madison, Wi, USA) was added to each well and the plate was incubated at 37 °C for 2 h. The absorbance was measured at 490 nm using a Synergy HT plate reader (BioTek, Winooski, VT, USA). Cell number was also directly counted under a phase-contrast microscope by using a blood cell counting chamber as needed.

### Immunoblotting

Vehicle or AP20187-treated cells were lysed using RIPA buffer containing 25 mM Tris HCl (pH 7.6), 150 mM NaCl, 1% sodium deoxycholate, 0.1% SDS and the protease inhibitor cocktail (Roche, Basel, Switzerland). Cell extracts with 5–20 μg of total protein were subjected to immunoblotting assays using primary antibodies against HA (3724, Cell Signaling Technology, Danvers, MA, USA), TNFAIP3 (sc-166,692, Santa Cruz Biotechnology, Dallas, TX, USA), E-cadherin (610,181, BD Biosciences, San Jose, CA, USA), N-cadherin (610,920, BD Biosciences), β-catenin (sc-7963, Santa Cruz Biotchnology), fibronectin (610,077, BD Biosciences), ERK1/2 (9102, Cell Signaling Technology), p-ERK1/2 (9101, Cell Signaling Technology), RSK1/2/3 (9355 s, Cell Signaling Technology), Phospho-p90 RSK (11,989 s, Cell Signaling Technology), GAPDH (2118 s, Cell Signaling Technology) and β-actin (A5441, Sigma-Aldrich, St., Louis, MO, USA). Appropriate horseradish peroxidase (HRP)-conjugated or fluorescence-labeled secondary antibodies (LI-COR Biosciences, Lincoln, NE, USA) were used to detect the primary antibodies bound to their antigens on the nitrocellulose membranes. The HRP activity was detected by using the ECL substrate solution (32,106, Thermo Fisher Scientific, Waltham, MA, USA), followed by exposure to X-ray film and quantified by the Odyssey Imaging System (LI-COR).

### Phalloidin staining

DCIS-iFGFR1 cells were cultured on cover slips placed in a 6-well plate, followed by AP20187 or vehicle treatment for 6 days. Cells were fixed for 15 min in 4% formaldehyde, and then washed three times in PBS. Cells were permeabilized in PBS containing 0.1% Triton X-100 for 5 min and washed 3 times with PBS. The prepared cells were stained in 1:20 dilution of Alexa Fluor® 488 Phalloidin (8878, Cell Signaling Technology) and 1:5000 dilution of DAPI in PBS for 15 min at room temperature. The stained cells were washed three times in PBS and dehydrated in serial ethanol solutions. After mounting the cover slip with stained cells onto glass slides, the stained cells were examined and imaged under a fluorescence microscope.

### RT-qPCR

Total RNA was extracted from cells by using Trizol reagent (Invitrogen). cDNA was synthesized by using a reverse transcription kit (Roche). TaqMan qPCR was performed in triplicates using a 7900 Real-time PCR machine (Applied Biosystems, Foster City, CA, USA). β-actin mRNA served as an internal control for gene expression. The average of delta Ct numbers was employed to calculate relative gene expression. The 5′ primers, 3′ primers and fluorescent probes matched from the Universal Probe Library (Cat. No. 04688970001, Roche) were: 5′-tgcacactgtgtttcatcgag, 5′-acgctgtgggactgactttc, Probe #74 for *TNFAIP3*; 5′-atcaggggccaggttttc, 5′-gggccaagcaccatctaat, Probe #13 for *PIM1*; 5′-ccagctgacaacaggaggag, 5′-cccatgagctccttgtacagat, Probe #3 for *SERPINE1*; 5′-ggccttgtgaacagatcagc, 5′-ctccggttcctgcacttg, Probe #69 for *FOSL1*; 5′-gtggacgggcagaatgtta, 5′-cgtggccagaatctccat, Probe #41 for *SDCBP2*; 5′-gctcctactgtgataagtccttcc, 5′-tgtcgcctgtgtggattct, Probe #10 for *ZNF362*; and 5′-tcccacccagaatctttaggta, 5′-gccggggttgagattcat, Probe #10 for *EHF*.

### RNA-Seq

DCIS-iFGFR1 cells (4.0 × 10^6^) were cultured in 10-cm plates overnight, treated with vehicle or 100 nM AP20187 for 3 and 16 h. Total RNA was extracted with Trizol reagent (Invitrogen) and subjected to RNA-Seq using Illumian HiSeqTM 2000. Biocomputational analysis was carried out to compare differential gene expression profiles induced by iFGFR1 activation at different time points. Differentially expressed genes were further analyzed by using the DAVID online analysis tool with the Gene Ontology (GO) and the Kyoto Encyclopedia of Genes and Genomes (KEGG) databases [46, 47]. *p* < 0.05 was used to select significant GO terms and KEGG pathways. The -log(*p* value) is the negative log10 of the *p* value.

### CRISPR/Cas9-based gene KO

To KO human *ERK1*, ERK,2 and *TNFAIP3* genes, gRNAs for each gene were designed using the Optimized CRISPR Design Tool as described previously [48]. Double-strand oligo DNA for each gRNA was cloned into the BbsI site of the SpCas9-2A-GFP plasmid (PX458, Addgene, Cambridge, MA, USA) for expressing sgRNA and Cas9. DCIS-iFGFR1 cells were transfected with the expression plasmids using Lipofectamin 2000. After 48 h, GFP-positive cells were sorted by flow cytometry and seeded in 96-well plates at an opportunity of 1 cell/well. Single cell clones were marked, amplified, and tested for gene expression by immunoblotting using antibodies against ERK1/2 or TNFAIP3. DNA samples of the candidate KO clones were prepared and sequenced to confirm the gene KO. Non-KO clones were used as control cells.

### Mammosphere growth assay

This assay was performed as described previously [49]. Briefly, an aliquot of 3000 cells in 100 μl of culture medium was added to each well of ultra-low attachment U bottom 96-well plates (Corning, Corning, NY, USA) to grow mammospheres. After culturing for 24 h, cells were treated with vehicle or 100 nM AP20187 for 7 days. Each treatment group had eight parallel samples. The cell spheres formed in each well were imaged and their diameters were measured using the Image Pro Plus 5.0 Software (Media Cybernetics, Rockville MD, USA).

### Xenograft tumor growth

Six- to 7-week-old BALB/c-nu mice were purchased from Beijing Huafukang Biosciences Inc., Beijing, China. DCIS-iFGFR1-Ctrl or DCIS-iFGFR1-TNFAIP3 KO cells were injected into each of the fourth pair mammary gland fat pads of these mice. After 3 days, mice were treated with AP20187 (1 mg/kg, 3 times/week, i.p.) or equal volume of solvent (< 50 μl). AP20187 was dissolved in ethanol at a stocking concentration of 10 mg/ml, and further diluted to 400 μg/ml in water solution of 10% PEG400 and 2% Tween-80 for injection. Tumor length and width were measured three times per week by using a caliper. Tumor volume was calculated by the formula: (length × width^2^) × π/6. Mice were sacrificed when the biggest tumor exceeded 1.5 cm in length. Tumors were harvested and weighed immediately.

### Hematoxylin and eosin (H&E) staining and immunohistochemistry

Collected xenograft tumor tissues were fixed in 4% paraformaldehyde, embedded in paraffin, and sectioned at a thickness of 5 μm. Sections were deparaffinized in xylene and rehydrated by going through ethanol series and water. Some sections were stained with H&E and used for histopathological examination. Other sections were soaked in 10 mM sodium citrate (pH 6.0) and heat-treated in a high-pressure cooker for 4 min. The section slides were washed in PBS and blocked in 5% bovine serum albumin (BSA) for 1 h. The prepared sections were incubated overnight at 4 °C with p-ERK1/2 antibody (4370 s, Cell Signaling Technology) at 1:400 dilution in PBS containing 5% BSA. After washing and incubation with biotinylated anti-rabbit IgG, the immunostaining signal was visualized with DAB kit (8059S, Cell Signaling Technology). The sections were counterstained with Harris Modified Hematoxylin, dehydrated and mounted with Permount for microscopy and imaging.

## Results

### Activation of iFGFR1 signaling in DCIS-iFGFR1 cells induces ERK1/2 phosphorylation, partial EMT, and cell proliferation

**Fig. 1:**
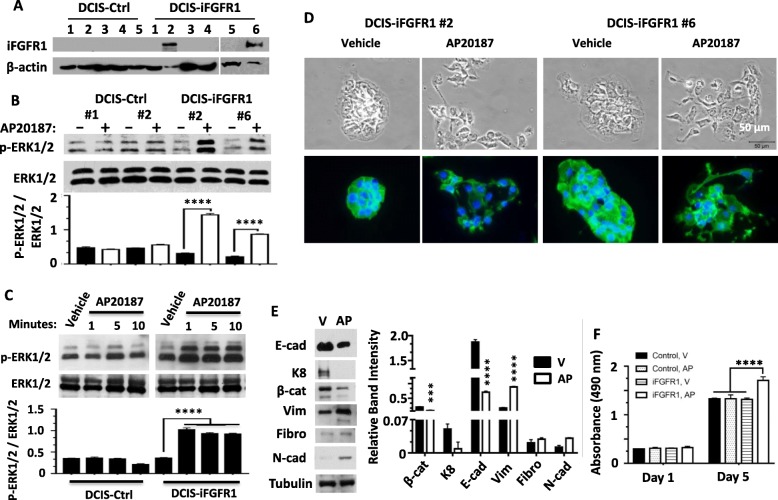
Activation of iFGFR1 induces ERK1/2 activation, cell morphological change, and cell proliferation. **a** Development of DCIS-Ctrl and DCIS-iFGFR1 cell lines. Cells expanded from single clones were assayed by immunoblotting with HA antibody. DCIS-Ctrl clones had no iFGFR1 expression. Two positive DCIS-iFGFR1 clones (#2 and #6) were detected. **b** AP20187 treatment had no effect on ERK1/2 in DCIS-Ctrl cell lines #1 and #2, but it increased p-ERK1/2 in DCIS-iFGFR1 cell lines #2 and #6 without affecting total ERK1/2 levels. The relative intensities of p-ERK1/2 to total ERK1/2 bands for each sample were calculated from three independent assays. **** *p* < 0.0001 by Student’s *t* test. **c** AP20187 treatment for the indicated time periods rapidly increased p-ERK1/2 in DCIS-iFGFR1 but not DCIS-Ctrl cells that were pre-cultured in serum-free medium for 12 h. **d** AP20187 treatment induced a fibroblast-like morphological change of both #2 and #6 DCIS-iFGFR1 cell lines. The *upper images* were recorded under a phase-contrast microscope. The *lower images* were recorded from phalloidin-stained cells pretreated with vehicle or AP20187 as indicated. **e** AP20187 (AP)-treated DCIS-iFGFR1 cells showed lower β-catenin, K8, and E-cadherin and higher vimentin, fibronectin, and N-cadherin when compared with vehicle (V)-treated DCIS-iFGFR1 cells. The relative band intensities shown in the bar graph were obtained from three independent assays. *** and **** *p* < 0.001 and 0.0001 by Student’s *t* test. **f** AP20187-activated iFGFR1 stimulated DCIS-iFGFR1 cell growth. DCIS-Ctrl and DCIS-iFGFR1 cells were treated with vehicle or AP20187 for 1 or 5 days as indicated. Cell viability was assayed from four independent samples by the CellTiter kit. Absorbance was measured at 490 nm. ***p < 0.001 by one-way ANOVA

To study the mechanism of FGFR1 signaling in human breast cancer progression, we generated DCIS-Ctrl control cell lines containing an empty vector and DCIS-iFGFR1 cell lines expressing the C-terminally HA-tagged iFGFR1 fusion protein (Fig. 1a). It has been shown that AP20187-induced dimerization of iFGFR1 resulted in the activation of the FGFR1 signaling [25, 35, 36]. After treatment with AP20187, both total ERK1/2 and p-ERK1/2 showed no changes in DCIS-Ctrl cells, while the levels of pERK1/2 were significantly increased in DCIS-iFGFR1 cell lines although total ERK1/2 levels remained the same (Fig. 1b). The high levels of p-ERK1/2 in DCIS-iFGFR1 cells were significantly induced by AP20187 within 1 min and could be maintained for hours (Fig. 1c and data not shown). Furthermore, the majority (89% ± 1.5%) of vehicle-treated DCIS-iFGFR1 cells formed epithelial colonies with tight cell-cell interactions, while only 20% ± 0.4% of AP20187-treated DCIS-iFGFR1 cells retained epithelial colony morphology and 80% ± 0.9% of these cells exhibited fibroblast cell morphology (Fig. 1d and data now shown). In the AP20187-treated cells, the epithelial markers E-cadherin, cytokeratin 8 (K8), and β-catenin were significantly reduced and the mesenchymal markers including vimentin, fibronectin, and N-cadherin were increased (Fig. 1e). Moreover, AP20187 treatment had no effect on DCIS-Ctrl cells but significantly increased the proliferation rates of DCIS-iFGFR1 cells (Fig. 1f). These results demonstrate that the AP20187-activated iFGFR1 is fully functional in terms of ERK1/2 activation, EMT induction, and cell proliferation.

### Activation of iFGFR1 signaling pathway changes the expression levels of important genes for regulating gene expression, cell proliferation, and cancer

**Fig. 2:**
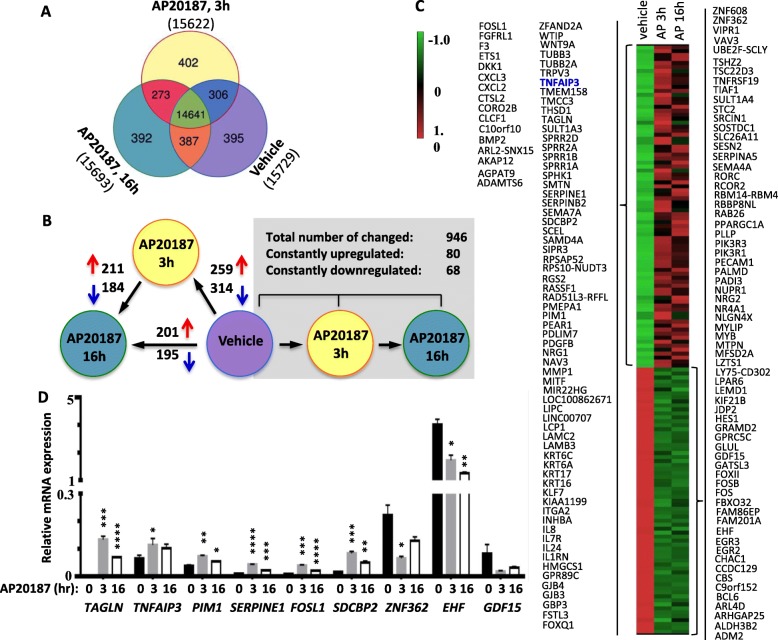
AP20187-induced changes of gene expression in DCIS-iFGFR1 cells identified by RNA-Seq. **a** Venn diagrams for the numbers of mRNAs detected in DCIS-iFGFR1 cells treated with vehicle, AP20187 for 3 h and AP20187 for 16 h as indicated. Three independent RNA samples were assayed by RNA-Seq in each group. Total number of mRNAs detected in each group and expression relationships among all three groups are indicated. **b** Comparison of AP20187-induced mRNA expression changes at different time points of treatment and identification of consecutively upregulated and downregulated mRNAs changed by AP20187 treatment. **c** Heatmap for the expression levels of the consecutively upregulated 80 genes and downregulated 68 genes. **d** Real-time RT-qPCR measurement of the indicated mRNA expression levels in DCIS-iFGFR1 cells with vehicle treatment (0) or AP20187 treatment for 3 or 16 h as indicated. *p < 0.05; **p < 0.01; ***p < 0.001; and ****p < 0.0001 by unpaired Student’s *t* test

To identify the genes regulated by the iFGFR1-signaling, we performed RNA-Seq analyses with nine RNA samples prepared from DCIS-iFGFR1 cells treated with vehicle (*n* = 3), AP20187 for 3 h (n = 3) or AP20187 for 16 h (n = 3). In general, more than 15,000 mRNA transcripts were detected in all three groups, and the expression levels of 6–7% of these transcripts were changed by AP20187 treatment (Fig. 2a). Specifically, when compared with vehicle treatment, AP20187 treatment for 3 h upregulated and downregulated mRNA expression of 259 and 314 genes, respectively (Fig. 2b and Additional file 1), and AP20187 treatment for 16 h upregulated and downregulated mRNA expression of 201 and 195 genes, respectively (Fig. 2b and Additional file 2). When compared between cells treated with AP20187 for 16- and 3-h, there were 211 upregulated and 184 downregulated genes (Fig. 2b and Additional file 3). After the overlapping mRNAs changed during 3 and 16 h of AP29187 treatment were filtered out, there were a total of 946 mRNAs that were either upregulated or downregulated (Fig. 2b). Eighty and 68 of the 946 mRNAs were consecutively upregulated and downregulated, respectively, at both 3- and 16-h time points of AP20187 treatment when compared with the vehicle-treated group (Fig. 2b and c). The remaining mRNAs were either upregulated at the 3-h time point and then downregulated at the 16-h time point or vice versa (Fig. 2b). RT-qPCR analysis validated all of the six selected mRNAs upregulated by AP20187 and two of the three selected mRNAs downregulated by AP20187 in DCIS-iFGFR1 cells. The remaining one showed a downregulation trend without reaching a significant level because of the larger expression variations in one group of samples (Fig. 2d). These results suggest that activation of the FGFR1-signaling pathway temporally regulates a subset of genes, which may reflect the functional complexity of the interactive networks involving the gene products regulated directly and indirectly by the FGFR1 signaling.

**Fig. 3:**
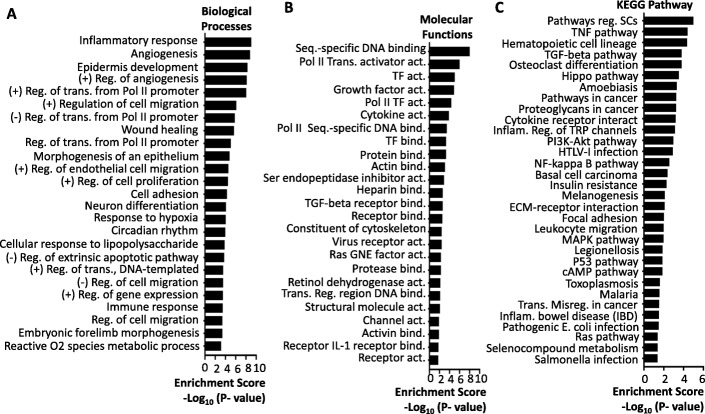
GO enrichment and KEGG pathway analysis of the FGFR1 signaling-regulated genes in DCIS-iFGFR1 cells. **a** GO analysis of the 946 AP20187-changed genes in DCIS-iFGFR1 cells identified 191 terms with significant gene enrichment based on biological processes (*p* < 0.05). The top 25 significantly enriched terms are shown here. **b** GO analysis of the 946 AP20187-regulated genes identified 38 terms with significant gene enrichment based on molecular functions (*p* < 0.05). The top 25 significantly enriched terms are listed. **c** Top 32 pathways identified by the KEGG pathway analysis of the 946 AP20187-changed genes in DCIS-iFGFR1 cells. *p* < 0.05 was used as a threshold to select significant GO terms and KEGG pathways. The -log(*p* value) is the negative log10 of the *p* value. *act.* activity, *bind.* binding, *GNE* guanyl-nuclotide exchange, *(+) Reg*. positive regulation, *(−) Reg*. negative regulation, *SCs* stem cells, *Seq.* sequence, *trans.* Transcription, *TF* transcription factor,

GO analysis of the differentially expressed 946 mRNAs upon AP20187 treatment revealed their enrichment in multiple biological processes such as inflammatory responses, angiogenesis, cell proliferation/migration/adhesion and gene regulation, and in multiple molecular functions including DNA binding, gene transcription, signaling protein-protein interaction, and Ras signaling (Fig. 3a and b). KEGG pathway analysis also indicates that the FGFR1-regulated genes are involved in pathways important for regulating stem cells, inflammation such as the TNF and NF-κB pathways, cancer growth and metastasis such as the NF-κB, hippo, PI3K-Akt, p53, and Ras pathways (Fig. 3c). These results suggest that the FGFR1 signaling pathway can promote EMT, cell growth, and carcinogenesis through regulating different genes involving multiple biological events and molecular signaling pathways.

### Activation of iFGFR1 signaling upregulates TNFAIP3 expression

**Fig. 4:**
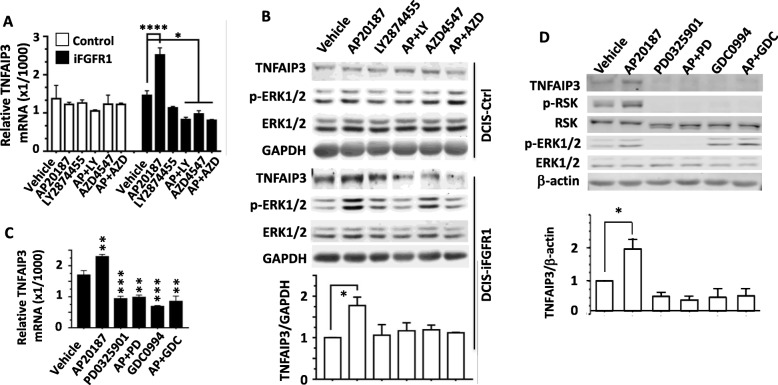
AP20187-induced TNFAIP3 expression is dependent on ERK1/2 activation in DCIS-iFGFR1 cells. **a** TNFAIP3 mRNA expression. DCIS-Ctrl (Control) and DCIS-iFGFR1 cells were treated with vehicle, FGFR1/2/3 inhibitor LY2874455 (LY, 500 nM) or FGFR1/2/3/4 inhibitor AZD4547 (AZD, 500 nM) as indicated for 1 h. Then, AP20187 (AP, 100 nM) was added to treat cells for another 6 h. TNFAIP3 mRNA was analyzed by real-time RT-qPCR and normalized to β-actin mRNA. Data were mean ± standard deviation (SD) of three independent experiments. * and ****p < 0.05 and 0.0001 vs. vehicle-treated group by one-way ANOVA. **b** Immunoblotting analysis. DCIS-Ctrl and DCIS-iFGFR1 cells were treated as described above for Panel A, except that cells were treated for another 24 h after adding AP20187. The ratios of TNFAIP3 band intensity to GAPDH band intensity were calculated from three repeating experiments. *p < 0.05 between vehicle- and Ap20187-treated groups by one-way ANOVA. **c** and **d** DCIS-iFGFR1 cells were treated with vehicle, MEK inhibitor PD0325901 (100 nM) or ERK1/2 inhibitor GDC0994 (1 μM) for 1 h, then AP20187 (100 nM) was added to treat the indicated cells for another 24 h. TNFAIP3 mRNA was analyzed by real-time RT-qPCR and normalized to β-actin mRNA. ** and ***p < 0.01 and *p* < 0.001 vs. vehicle-treated group by one-way ANOVA. (Panel C). Immunoblotting was performed by using antibodies against TNFAIP3, p-RSK, total RSK, p-ERK1/2, and total ERK1/2. The ratios of TNFAIP3 band intensity to β-actin band intensity were calculated from three repeating experiments. *p < 0.05 between vehicle- and AP20187-treated groups by one-way ANOVA; no significant differences between vehicle-treated group and all other inhibitor-treated groups (Panel D)

Among the genes consecutively upregulated by AP20187-activated iFGFR1, we were particularly interested in understanding how FGFR1 signaling regulates TNFAIP3 expression, since it plays an important role in NF-κB regulation but its role in breast cancer is unknown [37, 50]. AP20187-activated iFGFR1 robustly increased the expression of TNFAIP3 mRNA in DCIS-iFGFR1 cells, while this increase could be completely blocked by treating cells with FGFR inhibitors LY2874455 [51] and AZD4547 [52] (Fig. 4a). Accordingly, the TNFAIP3 and p-ERK1/2 protein levels were similar in DCIS-Ctrl cells treated with AP20187 or FGFR inhibitors, while the TNFAIP3 and p-ERK1/2 protein levels in DCIS-iFGFR1 cells were increased by AP20187 treatment and these increases were abolished by LY2874455 or AZD4547 treatment (Fig. 4b). Furthermore, the AP20187-induced TNFAIP3 mRNA and protein were positively associated with the increases in the phosphorylated active forms of ERK1/2 and/or p90-RSK. Inhibition of ERK1/2 activities by either ERK1/2 inhibitor GDC0994 or MEK inhibitor PD0325901 that prevents ERK1/2 activation abolished ERK1/2-mediated p90-RSK phosphorylation (activation), which also significantly reduced the basal and AP20187-induced levels of TNFAIP3 mRNA and protein (Fig. 4c and d). These results demonstrate that activation of the FGFR1 signaling pathway upregulates TNFAIP3 expression in an ERK1/2 activation-dependent manner.

### Upregulation of TNFAIP3 by iFGFR1 signaling is mainly dependent on ERK2 in DCIS-iFGFR1 cells

**Fig. 5:**
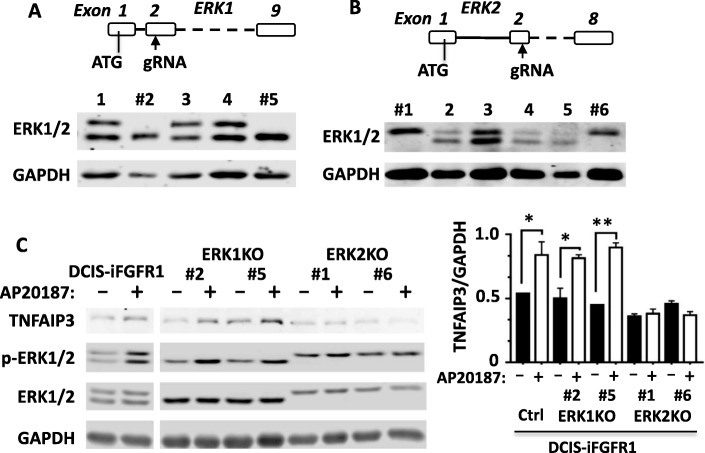
The effects of *ERK1* or *ERK2* KO on TNFAIP3 expression. **a** KO of *ERK1* by the CRISPR/Cas9 system in DCIS-iFGFR1 cells. A gRNA, 5’-CCACGUGCGCAAGACUCGCG, was designed based on the DNA sequence of human *ERK1* gene (NM_002746.2). This gRNA should guide Cas9 to cut the position in exon 2 of the human *ERK1* gene for coding the 60th amino acid (a.a.) residue. This strategy, if successful, disrupts the functions of all ERK1-splicing isoforms. Immunoblotting screening of 190 single clones identified eight KO clones, and the KO clones #2 and #5 are shown. **b** KO of *ERK2* in DCIS-iFGFR1 cells. A gRNA, 5’-UCUUUCAUUUGCUCGAUGGU, was designed based on the human *ERK2* DNA sequence (NM_002745.4). The Cas9-cutting site is corresponding to the coding sequence for the 90th a.a. residue in exon 2. This KO strategy disrupts all splicing isoforms of *ERK2*. Immunoblotting screening of 188 single clones identified six KO clones, and the KO clones #1 and #6 are shown. **c** DCIS-iFGFR1 control, ERK1 KO, and ERK2 KO cells were treated with vehicle (−) or AP20187 (+) for 24 h. TNFAIP3, p-ERK1/2, and total ERK1/2 were assayed by immunoblotting. GAPDH served as a loading control. The average ratios of TNFAIP3 band intensity to GAPDH band intensity were calculated from two independent assays. * and **p < 0.05 and *p* < 0.01 by one-way ANOVA

Although ERK1 and ERK2 share redundant functions, their specific roles have also been reported [53]. To define the specific roles of ERK1 and ERK2 in FGFR1-mediated TNFAIP3 expression, we co-expressed Cas9 with the sgRNA that specifically targets exon 2 of the human *ERK1* gene or the sgRNA that specifically targets exon 2 of the human *ERK2* gene in DCIS-iFGFR1 cells. Multiple KO cell lines for each gene were identified by screening individually isolated clones by PCR, followed by DNA sequencing (data not shown). Immunoblotting analysis confirmed the absence of the p44 ERK1 protein and the presence of the p42 ERK2 protein in the ERK1 KO cell lines and vice versa in the ERK2 KO cell lines (Fig. 5a and b). As indicated by the immunoblotting results of multiple experiments, KO of ERK1 or ERK2 did not change or only marginally increased the level of ERK2 or ERK1 (Fig. 5a–c). These KO cell lines showed normal growth in culture.

Again, AP20187 treatment induced TNFAIP3 protein expression in DCIS-iFGFR1 cells. Interestingly, AP20187 treatment also upregulated TNFAIP3 protein in two DCIS-iFGFR1 cell lines with ERK1 KO as it did in DCIS-iFGFR1 control cells with wild-type ERK1/2. However, AP20187 treatment failed to induce TNFAIP3 protein in two DCIS-iFGFR1 cell lines with ERK2 KO. Accordingly, AP20187 induced a more dramatic increase in ERK2 phosphorylation in ERK1 KO cells than ERK1 phosphorylation in ERK2 KO cells (Fig. 5c). These results demonstrate that TNFAIP3 expression stimulated by FGFR1 signaling is largely dependent on the activation of ERK2.

### TNFAIP3 is required for iFGFR1-mediated cell proliferation

**Fig. 6:**
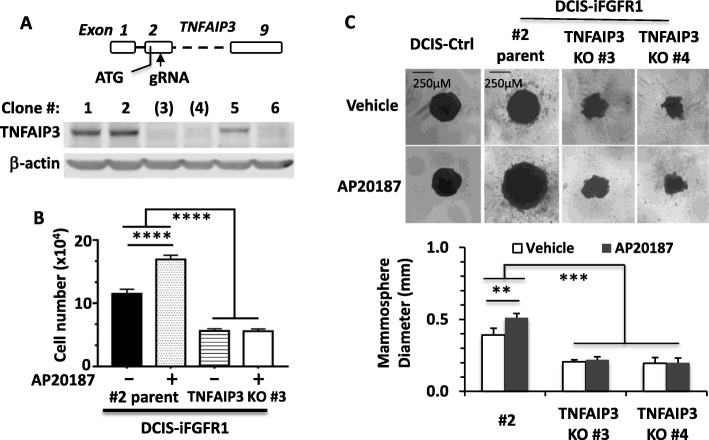
TNFAIP3 is required for FGFR1 activation-induced cell growth. **a** Generation of TNFAIP3 KO cell lines by the CRISPR/Cas9 system from DCIS-iFGFR1 cells. The gRNA 5’-UGCACCGAUACACACUGG was designed based on the human TNFAIP3 DNA sequence (NM_001270508.1) to guide Cas9 to cut the coding sequence for the a.a. residue 48 in exon 2. This targeting event disrupts the function of all three splicing variants of TNFAIP3. Immunoblotting screening of 100 individual clones identified six KO clones, and three KO clones (#3, #4 and #6) are shown. **b** KO of TNFAIP3 inhibited cell proliferation. DCIS-iFGFR1 #2 parent cells and TNFAIP3 KO #3 cells derived from the DCIS-iFGFR1 #2 parent cells were cultured in 6-well plate with 50,000 cells/well and treated with vehicle (DMSO) (−) or AP20187 (+) for 3 days before cells in each well were counted. Data are presented as average ± SD of six repeat assays in two independent experiments. ****p < 0.0001 by one-way ANOVA. **c** KO of TNFAIP3 inhibited mammosphere growth. The indicated cell lines were cultured in the U-bottom ultra-low attachment 96-well plates and treated with vehicle or AP20187 for 7 days. The DCIS-iFGFR1-#2 cell line is the parent cell line from which the TNFAIP3 KO #3 and #4 cell lines are derived. Representative images of the spheres formed were shown. The mean of the sphere diameters for each group was calculated from eight replicates. The experiment was repeated three times. ** and ***p < 0.01 and *p* < 0.001 by one-way ANOVA

To address whether TNFAIP3 is required for FGFR1-mediated cell proliferation, we co-expressed Cas9 with the sgRNA that targets the second exon of the human *TNFAIP3* gene. Our screening identified several KO clones and we used two of these clones for experiments (Fig. 6a). As expected, AP20187 treatment significantly increased the proliferation rate of the DCIS-iFGFR1 #2 parent control cells. Interestingly, TNFAIP3 KO cells derived from the DCIS-iFGFR1 parent cells failed to respond to AP20187 treatment in terms of cell proliferation, indicating that TNFAIP3 is required for iFGFR1-mediated cell proliferation. Furthermore, KO of TNFAIP3 inhibited cell proliferation when compared with their parent control cells in the absence of AP20187 treatment, suggesting that TNFAIP3 may also be required for the endogenous FGFR1-mediated cell growth or involved in other cell growth pathways. (Fig. 6b). Consistent results were also obtained from assaying the growth of three-dimensional (3D) mammospheres. DCIS-Ctrl cells formed medium-sized spheres that were insensitive to AP20187 treatment. The DCIS-iFGFR1 #2 parent control cells formed medium-sized spheres in the absence of AP20187, while AP20187 treatment significantly increased the sphere sizes formed from these cells. However, both #3 and #4 lines of the TNFAIP3 KO DCIS-iFGFR1 cells only developed small spheres and AP20187 treatment was unable to enhance their growth (Fig. 6c). These results indicate that TNFAIP3 is essential for FGFR1 signaling-stimulated cell proliferation and mammosphere growth.

### TNFAIP3 is required for the iFGFR1 signaling pathway-promoted tumor growth in mice

**Fig. 7:**
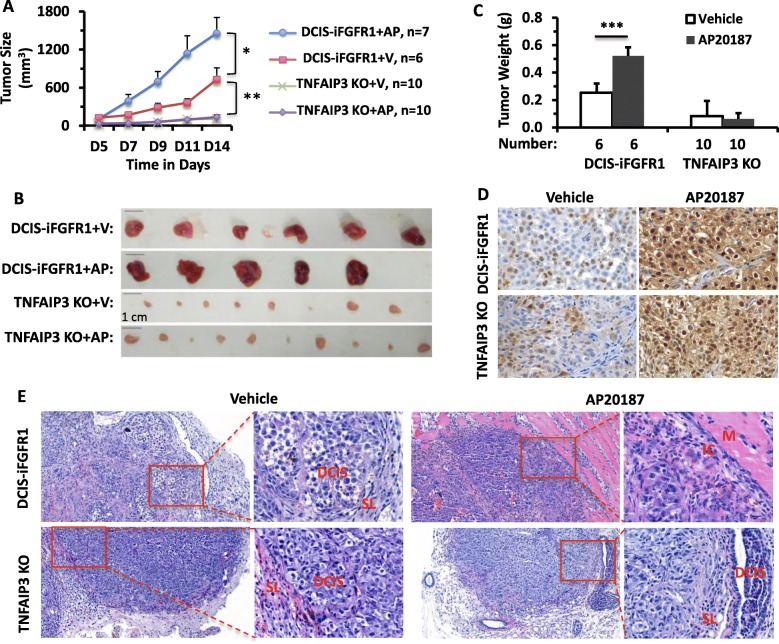
TNFAIP3 KO inhibits DCIS-iFGFR1 cell-derived xenograft tumor growth and progression in mice. **a** Tumor growth curves. Two million DCIS-iFGFR1 or TNFAIP3 KO DCIS-iFGFR1 cells were injected into one of the fourth pair mammary gland fat pads of nude mice on day 1. Six to ten mice in each group were treated with vehicle (V) or AP20187 (AP) as indicated from day 4 to day 14. Tumor volume was measured as described in the Methods section and presented as mean ± SD. * and **p < 0.05 and *p* < 0.01. **b** Images of individual tumors derived from the indicated cells in mice treated with vehicle or AP20187 as indicated. **c** Average weights of wet tumors collected from mice shown in Panel A on day 14. The number of tumors weighed in each group is indicated. ***p < 0.001 by unpaired Student’s *t* test**. d** Immunohistochemical staining for p-ERK1/2 (*brown color*) in the tissue sections prepared from vehicle or AP20187-treated DCIS-iFGFR1 control and TNFAIP3 KO xenograft tumors. **e** H&E-stained tissue sections prepared from vehicle- or AP20187-treated DCIS-iFGFR1 control and TNFAIP3 KO xenograft tumors. The *boxed areas* are also shown in higher magnification as indicated. *DCIS* ductal carcinoma in situ-like area, *IC* invasive carcinoma-like area, *M* skeletal muscle area, *SL* surrounding stromal cell layer

To investigate the role of TNFAIP3 in FGFR1-promoted breast tumor growth in vivo, we injected DCIS-iFGFR1 #2 parent control cells and TNFAIP3 KO DCIS-iFGFR1 cells into the fat pads of nude mouse mammary glands. DCIS-iFGFR1 cells formed tumors and the average tumor weight reached about 0.25 g in 14 days. AP20187 treatment of mice markedly accelerated tumor growth, which increased the average tumor weight to about 0.5 g in 14 days. Interestingly, TNFAIP3 KO DCIS-iFGFR1 cells only grew very small tumors either with or without AP20187 treatment. Their average tumor weight was less than 0.08 g on day 14 after the same number of cells was injected into the mammary fat pads of nude mice (Fig. 7a–c). In both vehicle-treated DCIS-iFGFR1 and TNFAIP3 KO xenograft tumors, p-ERK1/2 signals were only detected in a subset of tumor cells and these immunostaining signals were relatively weak. In contrast, in both AP20187-treated DCIS-iFGFR1 and TNFAIP3 xenograft tumors, p-ERK1/2 immunostaining signals were detected in almost all of the tumor cells at stronger levels (Fig. 7d). In both types of vehicle-treated tumors, the tumor cells grew in clusters and each cell cluster was surrounded by multiple layers of stromal cells, which simulates the DCIS lesion morphologies. However, in AP20187-treated DCIS-iFGFR1 tumors, the tumor cell morphology exhibited much higher degrees of heterogeneity and invasiveness. Some tumor cells had invaded the skeletal muscle tissue. In the AP20187-treated TNFAIP3 KO tumors, most tumor areas displayed similar morphologies observed in the vehicle-treated TNFAIP3 KO tumors. In certain areas, highly differentiated DCIS-like structures were also observed (Fig. 7e). These results demonstrate that TNFAIP3 is not required for iFGFR1-mediated ERK1/2 activation, but it is essential for iFGFR1-induced DCIS-iFGFR1 tumor growth and progression in vivo.

## Discussion

FGFR1 signaling is known to activate ERK1/2 MAPKs to regulate cell growth, differentiation and transformation [54]. Although FGFR1 signaling pathways have been well studied, some key questions remain unaddressed. For example, it is not easy to discern the functional specificity of ERK1 from ERK2 in mediating the FGFR1 signaling to the downstream signaling components because of their significant functional redundancy. In addition, the FGFR1 signaling-regulated genes important for cell proliferation and carcinogenesis are still largely undefined. In this study, we established DCIS-iFGFR1 cell lines in which iFGFR1 activation is induced by AP20187 treatment. We demonstrated that activated iFGFR1 activates ERK1/2, induces partial EMT, and increases cell proliferation, which is consistent with the results reported previously [25, 35, 36]. Using this cell system, we characterized the genes regulated by FGFR1 signaling. We found that activation of iFGFR1 changed the expression levels of 946 genes. These genes exhibited several expression patterns: the expression levels of 80 genes were consecutively increased while the expression levels of 68 genes were consecutively decreased during both short-time (3 h) and long-time (16 h) activation of the iFGFR1 signaling; a subset of genes were upregulated at the 3-h time point but downregulated at the 16-h time point; and another subset of these genes were downregulated at the 3-h time point but upregulated at the 16-h time point. These complex gene regulatory patterns by the activated iFGFR1 signaling suggest that the FGFR1 signaling pathway can directly activate and suppress gene expression, as well as, potentially regulate the expression of many other genes through its directly regulated gene products. Understanding this FGFR1-regulated gene network will help to identify downstream targets of the FGFR1 signaling pathway.

In agreement with the role of FGFR1 in promoting cell proliferation and carcinogenesis, a number of iFGFR1-upregulated genes are cancer-driving genes such as ETS1, PIM1, NRG1, MMP1, and FOXQ1, while some iFGFR1-downregulated genes are tumor suppressors such as NR4A1 and GDF15. However, it is currently unclear why the activated FGFR1 signaling also downregulates some growth-promoting genes such as EGR2/3, MYB, and PIK3R1/3 (Fig. 2c). Bioinformatic analysis of the FGFR1 signaling-regulated genes revealed that these genes are involved in many biological processes, molecular functions, and signaling pathways, including cell proliferation, adhesion and migration, gene regulation, basal cell carcinogenesis, as well as general cancer-promoting pathways such as MAPK, Hippo, PI3K-AKT, Ras, p53, and NF-κB pathways (Fig. 3). These results suggest that the downstream molecular mechanisms responsible for mediating FGFR1 function are through coordinating multiple signaling pathways that govern cell proliferation, behavior and differentiation.

Among the iFGFR1-upregulated genes, we further studied the role of TNFAIP3 in FGFR1 signaling-promoted DCIS.COM cell growth. We demonstrated that activation of iFGFR1 robustly upregulates TNFAIP3 mRNA and protein in DCIS-iFGFR1 cells and this upregulation can be completely blocked by either FGFR inhibitors or ERK1/2 inhibitors. Furthermore, KO of ERK2 completely abolished the FGFR1 signaling-induced TNFAIP3 upregulation, while KO of ERK1 showed little effect on TNFAIP3 expression. These findings identified a gene expression-regulatory axis of FGFR1-ERK2-TNFAIP3. Our data also showed that in both 2D culture and 3D mammosphere growth assays, activation of the iFGFR1 signaling increased the growth of DCIS-iFGFR1 cells, while KO of TNFAIP3 in these cells completely diminished the iFGFR1 signaling-induced cell growth. Consistent results were also observed in the xenograft tumor growth assay in mice, where activation of the iFGFR1 signaling markedly enhanced tumor growth and KO of TNFAIP3 inhibited tumor growth derived from DCIS-iFGFR1 cells. Interestingly, KO of TNFAIP3 not only abolished cell, mammosphere, and tumor growth induced by AP29187-activated iFGFR1, but also reduced cell, mammoshpere, and tumor growth in the absence of AP29187 treatment when compared with the tumors derived from DCIS-iFGFR1 cells with wild-type *TNFAIP3*. This may be explained by the role of TNFAIP3 in mediating the cell growth function of the endogenous FGFRs and/or the additional functions of TNFAIP3 involved in other cell growth-promoting pathways. In summary, our results indicate that TNFAIP3 is essential for FGFR1 signaling-induced breast cancer cell growth in culture and tumor growth in vivo.

Histopathological examination of the xenograft tumors revealed that activation of the iFGFR1 signaling promoted DCIS.COM tumor progression to invasive cancer. Interestingly, TNFAIP3 KO DCIS.COM xenograft tumors were insensitive to iFGFR1 activation induced by AP20187. These KO tumors exhibited mostly DCIS morphology. These results suggest that FGFR1 signaling can strongly promote DCIS progression to invasive cancer and TNFAIP3 is an essential contributing factor in this process.

The active mutants of HRAS, KRAS, and NRAS were found in a subset of breast cancers [55]. Although active Ras mutations and FGFR1 amplification and overexpression barely occur in the same breast cancer cells, FGFs are always present in the tumor microenvironment and FGFRs are expressed in breast epithelial and cancer cells. It is unknown whether FGFR1 activation can further activate downstream signaling in breast epithelial and tumor cells with an active Ras mutation to promote these cell growth and progression to a more aggressive cancer cell phenotype. MCF10AT cells are derived from mutant H-Ras transfected MCF10A normal cells and the initial transplantation of MCF10AT cells only develop differentiated ducts in mice. DCIS.COM cells are derived from passaged MCF10AT xenograft growth and DCIS.COM cells mainly form non-invasive DCIS tumors [5–8]. These findings suggest that expression of mutant H-Ras in these cells is insufficient to promote invasive cancer cells. In our study, iFGFR1 activation further increases ERK1/2 activity in DCIS.COM cells, accelerates their proliferation in culture and promotes their tumor growth and progression to invasive cancer in vivo. Our results indicate that FGFR1 activation has an additive role to mutant H-Ras in promoting DCIS cell growth and progression. It has been reported that wild-type H- and N-Ras promote mutant K-ras-driven tumorigenesis [56]. It is possible that activation of FGFR1 activates the endogenous Ras proteins in DCIS.COM cells, which cooperate with mutant H-Ras to promote breast cancer cell proliferation, and progression. Alternatively, FGFR1 activation may also work with mutant Ras to promote breast cancer cell proliferation and progression via its other signaling pathways that do not use Ras and ERK1/2. Importantly, KO of TNFAIP3 inhibited tumor growth promoted by both mutant H-Ras and FGFR1 activation, suggesting that TNFAIP3 may serve as a potential target for inhibiting ER- breast cancer with active mutant Ras and/or active FGFR1 signaling.

## Conclusions

Activation of FGFR1 signaling in DCIS.COM cells induces ERK1/2 activity, EMT, and cell proliferation in culture and promotes cell-derived xenograft tumor growth and progression to invasive cancer in mice. Activation of FGFR1 signaling upregulates and downregulates many genes. FGFR1 signaling upregulates TNFAIP3 expression via activating ERK2. TNFAIP3 expression is required for FGFR1 signaling-promoted DCIS.COM cell proliferation, mammosphere growth, tumor growth and progression.

## Additional files

Additional file 1: Differentially expressed genes identified in DCIS-iFGFR1 cells treated with AP20187 or vehicle for 3 h. (XLS 365 kb)

Additional file 2: Differentially expressed genes identified in DCIS-iFGFR1 cells treated with AP20187 or vehicle for 16 h. (XLS 273 kb)

Additional file 3: Differentially expressed genes identified in DCIS-iFGFR1 cells treated with AP20187 for 16 or 3 h (XLS 259 kb)

## Abbreviations

DCIS: Ductal carcinoma in situ
EMT: Epithelial-to-mesenchymal transition
ERK1/2: Extracellular-signal regulated kinases 1 and 2
ER: Estrogen receptor
FGF: Fibroblast growth factor
FGFR: Fibroblast growth factor receptor
HER2: Human epidermal growth factor receptor 2
KO: Knockout
